# “Too much guts and not enough brains”: (epi)genetic mechanisms and future therapies of Hirschsprung disease — a review

**DOI:** 10.1186/s13148-019-0718-x

**Published:** 2019-09-13

**Authors:** Emilie G. Jaroy, Lourdes Acosta-Jimenez, Ryo Hotta, Allan M. Goldstein, Ragnhild Emblem, Arne Klungland, Rune Ougland

**Affiliations:** 1 Clinic for Diagnostics and Intervention and Institute of Medical Microbiology, Oslo University Hospital, Rikshospitalet, 0027 Oslo, Norway; 2 Department of Pediatric Surgery, Oslo University Hospital, Rikshospitalet, 0424 Oslo, Norway; 3 Department of Pediatric Surgery, Massachusetts General Hospital, Harvard Medical School, Boston, MA USA; 4 Faculty of Medicine, Institute of Clinical Medicine, University of Oslo, 0317 Oslo, Norway; 5 Faculty of Medicine, Institute of Basic Medical Sciences, University of Oslo, 0317 Oslo, Norway; 6 Department of Surgery, Baerum Hospital, Vestre Viken Hospital Trust, 3004 Drammen, Norway

**Keywords:** Enteric nervous system, Epigenetics, Development, HSCR, Hirschsprung, Neural crest, Cell therapy

## Abstract

Hirschsprung disease is a neurocristopathy, characterized by aganglionosis in the distal bowel. It is caused by failure of the enteric nervous system progenitors to migrate, proliferate, and differentiate in the gut. Development of an enteric nervous system is a tightly regulated process. Both the neural crest cells and the surrounding environment are regulated by different genes, signaling pathways, and morphogens. For this process to be successful, the timing of gene expression is crucial. Hence, alterations in expression of genes specific for the enteric nervous system may contribute to the pathogenesis of Hirschsprung’s disease. Several epigenetic mechanisms contribute to regulate gene expression, such as modifications of DNA and RNA, histone modifications, and microRNAs. Here, we review the current knowledge of epigenetic and epitranscriptomic regulation in the development of the enteric nervous system and its potential significance for the pathogenesis of Hirschsprung’s disease. We also discuss possible future therapies and how targeting epigenetic and epitranscriptomic mechanisms may open new avenues for novel treatment.

## Background

Epigenetics and epitranscriptomics (i.e., modifications of macromolecules like DNA and RNA) are known to be of crucial importance in the development of a central nervous system. Yet, very little is known about the role of these mechanisms in the differentiation of neural crest derivatives and development of a functional enteric nervous system. In the following, we describe how modulation of epigenetic and epitranscriptomic pathways may open for novel treatment of human neurocristopathies by targeting these mechanisms.

## Introduction

Hirschsprung disease (HSCR) is a congenital anomaly and the most common enteric neuropathy. It affects about 1.43/10,000 [1] and is characterized by lack of ganglion cells (aganglionosis) in a segment of the distal bowel. The aganglionic segment invariably involves the internal anal sphincter and extends proximally to affect a variable extent of the colon. The absence of enteric neurons leads to a tonic contraction of the affected segment, resulting in gastrointestinal obstruction. The condition is usually symptomatic in the neonatal period. The treatment of HSCR is surgical excision of the aganglionic bowel and a coloanal anastomosis. However, even with the best available therapy, there is significant long-term morbidity associated with the condition [2, 3]. Fecal incontinence and constipation are common [4].

The genetic background of HSCR is complex. The majority of HSCR cases are sporadic (80%) while the rest are familial. Furthermore, there is a 4:1 predominance in males, with the estimated RET mutation penetrance of 72% in males and 51% in females [5]. The male to female ratio is significantly higher for short-segment HSCR disease in comparison with long-segment HSCR disease [5, 6]. Although most cases occur as an isolated trait (70%), 12% of patients have an associated chromosomal abnormality, and the majority of those have trisomy 21. In addition to trisomy 21, HSCR disease is associated with a wide range of congenital anomalies and syndromes such as distal limb, craniofacial, central nervous system, genital, kidney, and cardiac malformations; Mowat-Wilson; Goldberg-Shprintzen; Shah-Waardenburg; and congenital central hypoventilation syndrome. Syndromic HSCR shows a Mendelian inheritance, while non-syndromic HSCR displays a non-Mendelian inheritance with a low sex-dependent penetrance and variable expression [7]. This is supportive of the hypothesis of HSCR being a multigenic disorder, implicating that one or more genes with low penetrance are involved. In addition, mutations in more of the HSCR-associated genes are hypothesized to result in a more severe phenotype, i.e., a longer length of the intestine is affected.

**Fig. 1:**
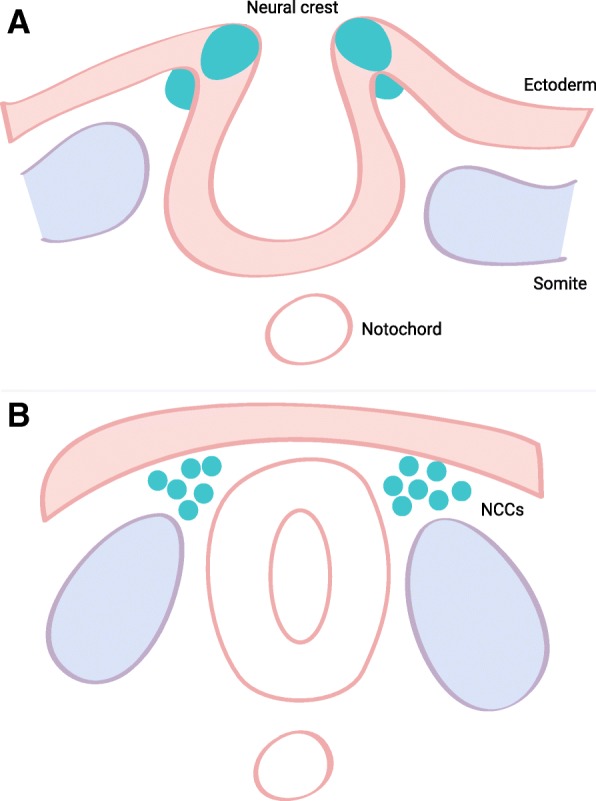
**a**, **b** ENS development. Ganglion cells of the ENS are derived from neural crest cells (NCCs). NCCs arise from the embryonic ectoderm cell layer

**Fig. 2:**
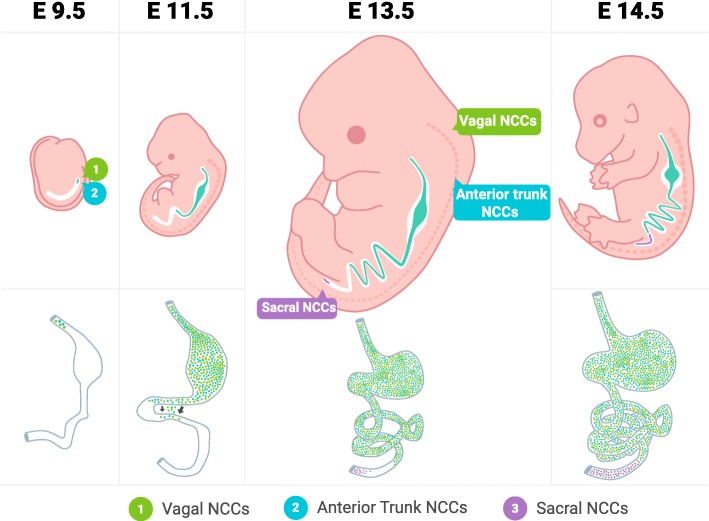
ENS development. In mice, ENS development has been studied thoroughly. Vagal NCCs migrate in a rostral to caudal direction eventually colonizing the entire length of the gut. Vagal NCCs invade the anterior foregut and continue along the midgut and hindgut. On embryonic day 11.5, there is a small wave of NCCs that cross over from the foregut to the hindgut. There is also a minor contribution of anterior trunk NCCs to the foregut and of sacral NCCs to the hindgut

The ganglion cells of the enteric nervous system (ENS) are entirely derived from the neural crest which is a transient, multipotent cell population originating from the neural tube [8, 9]. Neural crest cells originate at different axial levels (cranial, cardiac, vagal, trunk, and sacral) and migrate extensively throughout the embryo to colonize multiple organ primordia and differentiate into a variety of cell types and tissues [10]. The ENS is mainly derived from vagal neural crest cells, with a minor contribution from the sacral neural crest. Around the third week of human pregnancy, the vagal neural crest cells proliferate and invade the anterior foregut at which point they are referred to as enteric neural crest-derived cells (ENCDCs). During the following weeks, the ENCDCs migrate in a rostral to caudal direction eventually colonizing the entire length of the gut around week 7. Simultaneously, the migrating ENCDCs differentiate into defined enteric neurons and glia [11, 12]. The exact mechanisms orchestrating this journey of the vagal neural crest cells are not well characterized. However, it is known that signaling pathways, particularly the RET/GFRα1/GDNF and EDNRB/ECE1/EDN3 pathways, the transcription factors SOX10 and PHOX2B, and a number of morphogens such as netrin, sonic hedgehog, or semasphorins are of crucial importance [12–16] (Figs. 1 and 2).

Several genes are associated with ENS development, thereby also in the pathogenesis of HSCR disease. RET is identified as the main HSCR gene as the RET mutation is found in 50% of familial and 15–20% of sporadic HSCR cases [5]. RET is a transmembrane receptor tyrosine kinase which, upon binding of its ligand, glial-derived neurotrophic factor (GDNF), activates a variety of downstream pathways. Extensive reviews summarize the genes that have been associated with HSCR, and new genes are added to this list constantly [5, 17–21]. Recently, four de novo mutations were identified by whole exome sequencing: DENND3, NCLN, NUP96, and TBATA [22]. Even if it seems likely that somatic mutations that occur during ENS development contribute to HSCR pathogenesis, the evidence so far is inconclusive. Hence, the exact contribution of ENS-specific genes and their interplay in the pathogenesis of HSCR remains elusive.

## Epigenetic regulation

All nuclear cells of the human body contain the same sequence of DNA, the genome, yet they display very diverse cell-specific functions. To achieve differentially expressed genes without altering the genome sequence, nature has evolved an intricate system of epigenetic regulation. DNA methylation and histone modifications are most studied. These two epigenetic processes are partly linked and have critical roles for embryonic development and neurogenesis [23]. Various RNA molecules make up a third, and more heterogeneous group of epigenetic modifiers. Such RNA modifications are referred to as epitranscriptomics. Hence, epigenetics refers to functional alterations of the genome without a change in the nucleotide sequence, and epitranscriptomics refers specifically to functional alterations in the transcriptome without a change in the ribonucleotide sequence. Recently, the reversible nature of regulatory RNA modifications was discovered, and due to its recent discovery, this has not yet been extensively studied [24, 25]. However, an emerging body of evidence suggests that such modifications play important roles in neurogenesis and embryonic development [26–28]. Here, we review the current knowledge of epigenetic and epitranscriptomic regulation in the development of the ENS and its potential significance for the pathogenesis of HSCR. We also discuss possible future therapies and how targeting of epigenetic and epitranscriptomic mechanisms may open new approaches for novel treatment.

## DNA methylation and demethylation

**Fig. 3:**
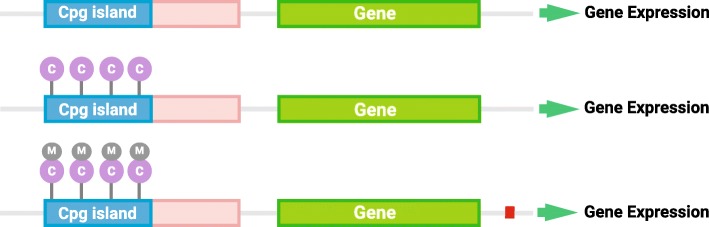
**a**, **b** DNA methylation. In the promoter area of genes, hypomethylation of the CpG island increases gene expression, while hypermethylation of the CpG island reduces gene expression. Hence, DNA hypomethylation activates genes while hypermethylation silences them

**Fig. 4:**
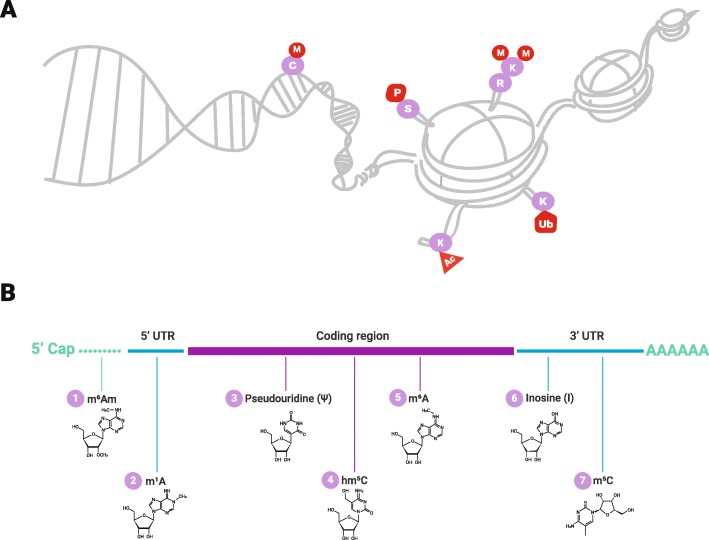
Epigenetic regulation. **a** DNA methylation; methyl groups attach to cytosine residues on the DNA strand. Histone modifications; the DNA strand wraps around an octamer of four core histones to form a nucleosome. Each of the histone proteins has characteristic side chains or tails enriched in lysine and arginine residues. These side chains and tails can be modified post-translationally, modifications such as methylation, ubiquitination, acetylation, and phosphorylation. **b** mRNA modifications either occur in the 5′cap, in the coding region, or in the 3′ or 5′ untranslated region. Chemical modifications in mRNA are illustrated in the figure; N^6^, 2′-O-dimethyladenosine (m^6^Am); N1-methyladenosine (m^1^A); Pseudouridine (Ψ); 5-hydroxylmethylcytidine (hm^5^C); N6-methyladenosine (m^6^A); Inosine (I); and 5-methylcytidine (m^5^C)

DNA methylation is the addition of a methyl group to the DNA strand (Fig. 4). This addition is performed by methyltransferases (DNMTs) and recruits methyl-binding proteins, e.g., MeCP2. Hence, DNMTs and MeCP2 are essential for normal mammalian development as they regulate gene expression, X-chromosome inactivation, genomic imprinting, and genomic stability by determining the methylation degree of the genome [29, 30]. Hypermethylation silences genes, while hypomethylation increases transcription (Fig. 3). Some DNMTs establish the initial DNA methylation patterns (DNMT1), and others maintain DNA methylation over cell generations (DNMT2 and DNMT3). MeCP2 is necessary to bind methyl groups on the DNA strand. Hence, DNMTs and MeCP2 regulate ENS development through the DNA methylation of genes involved in neurogenesis. This suggests that an aberrant methylation pattern may lead to an unfavorable increase or decrease of gene expression, which may contribute to HSCR [31, 32].

Both DNMT3B and MeCP2 expression are decreased in neural stem cells obtained from HSCR patients, which result in a decrease of global DNA methylation. This may contribute to an aberrant expression pattern of HSCR-associated genes [32, 33]. Target genes of DNMT3B have been identified, and their expression patterns analyzed and compared in HSCR patients versus controls. These target genes were upregulated in HSCR patients, which is consistent with the lower global DNA methylation due to downregulation of DNMT3B described in these patients [34]. Moreover, knockdown of DNMT3B in human embryonic stem cells leads to hypomethylation that consequently increases the expression of neural crest-specific genes (Pax3, Pax7, FoxD3, Snail2, and Sox10), and accelerates neural crest differentiation [35]. In addition, the expression of MeCP2 is lower in HSCR patients compared with controls [33]. This demonstrates that both DNMTs and MeCP2 contribute to HSCR pathogenesis by regulating gene expression [34].

Several HSCR-associated genes are regulated by the methylation degree of their promoter areas. One of these genes is RET, and it has been suggested that the level of RET expression determines the length of the aganglionic segment [36]. The RET-promoter has a 5′CpG3′ rich region highly susceptible for methylation, and its methylation degree regulates RET expression. Epigenetic inactivation by promoter hypermethylation of RET has been demonstrated [32]. In addition, the expression of GDNF (the ligand of RET) is decreased in some HSCR patients due to a promoter area hypermethylation [37]. Interestingly, demethylation of GDNF promotes cell proliferation and viability, cell cycle progression, and cell invasion in studies in vitro of cells derived from HSCR tissues [37]. EDNRB, another important gene in HSCR pathogenesis, is overexpressed in some HSCR patients when compared with controls, and it has a hypomethylated promotor area [38]. Furthermore, an aberrant methylation pattern has been found in the promoter area of PHOX2B in patients with neuroblastoma tumors, tumors that originate from neural crest cells [32]. This finding supports the hypothesis that the methylation degree of PHOX2B may also play a role in the pathogenesis of neurocristopathies, such as HSCR. Thus, aberrant methylation patterns resulting in epigenetic inactivation or overactivation of HSCR-associated genes are implicated in the development of HSCR [32].

## Histone modifications and chromatin

Histone modifications and chromatin-associated protein complexes are crucially involved in the control of gene expression and the determination of cell fate, especially during development. The role of histone modifications is of critical importance in CNS development. ENS development may be regulated by the same mechanisms, and if that is the case, then HSCR pathogenesis could be linked to histone modifications [39].

DNA is a very long molecule that requires tight packaging. There are architectural proteins that have evolved for this purpose, such as HU proteins and histones. Histones package and compact eukaryotic DNA by assembling into nucleosome core particles. Nucleosomes are complex structures and the fundamental unit of chromatin. They are formed by an octamer of four core histones (H3, H4, H2A, H2B) wrapped almost twice by 147 base pairs of DNA. Each of the histone proteins has a characteristic side chain or tail rich in lysine and arginine residues, which can have a large number of post-translational modifications, including methylation/demethylation, acetylation/deacetylation, phosphorylation, ubiquitination, and sumoylation (Fig. 4). This affects the chromatin structure and consequently gene expression and cellular phenotype [34, 39–41]. Two major forms of chromatin exist, silent (closed) heterochromatin and active (open) euchromatin. The two different forms are characterized by a certain subset of histone marks.

Pluripotent embryonic stem cells have distinct epigenetic features, such as an enrichment of histone modifications related to active chromatin [42, 43]. In order to maintain the stem cell state, a particular subset of genes is expressed while another subset is silenced. However, upon differentiation, the gene expression profile must be rapidly changed; hence, certain genes are kept in a “standby” state by a “dual labeling” of both activating and repressive histone marks, the so-called bivalent domains [39, 44]. These bivalent domains keep the genes silent but poised for immediate action, allowing timely activation once differentiation is induced [39, 41, 43, 44]. Yet, bivalent promoters are not restricted to developmentally regulated genes. Bivalency is complex and extends to different gene families in several different cell types [41].

Histone methylation and demethylation occurs on both lysine and arginine residues on histone tails. Histone methyltransferases (HMTs) add methylation marks, histone demethylases remove them. Many promoters in enteric stem cells (ESCs) are marked with both activating and repressive histone modifications, such as the activating H3Kme4 mark and the repressive H3K27me3 mark. H3K4 methylation and its maintenance have been found to participate in the development, pluripotency, and early ESC differentiation [41]. Moreover, it has been found that the demethylase JMJD2A modulates H3K9me3 of neural crest cells and thus allow neural crest cell specification to occur [32].

Histone acetylation is regulated by two groups of enzymes with opposing actions, histone acetyltransferases (HATs), and histone deacetylases (HDACs). They both hook onto a group of lysine side chains on histones. HATs neutralize lysine’s positive charge, thereby weakening the interaction between the DNA strand and the histones. Hence, they function as transcriptional co-activators. HDACs reverse lysine acetylation, restore its positive charge, and stabilize chromatin architecture. Hence, HDACs function as transcriptional repressors [44]. There are many examples of the correlation between the development of the ENS and HDACs. HDACs are essential in controlling neural crest migration [45, 46]. For later stages of ENS development, HDAC4 is required for differentiation of neural crest-derived cells [47]. Hence, HDAC4 is associated with neural crest-related diseases and syndromes. HDAC1 controls ESCs differentiation, and in animal models, it has been found to play an important role in ENS development [48, 49]. Furthermore, HDAC1 and HDAC2 are able to bind to promoter regions and promote differentiation of NCCs to peripheral glia [50]. Also, HDAC3 and HDAC8 are found to be essential for neural crest development [51].

Histone modifications enhance or silence transcription of specific genomic regions and may also apply to HSCR-associated genes such as RET. For example, the protein complex HOXB5 regulates RET by altering chromatin conformation. Hence, a dysregulation of RET expression by HOXB5 could result in insufficient RET expression and Hirschsprung disease [52]. Thus far, little is known about the molecular basis of gene expression regulation of HSCR-associated genes. If it is shown that the expression of specific genes affects HSCR susceptibility, they could potentially be corrected [53].

## Epitranscriptomics and microRNAs

Transcriptomics is defined as the study of the transcriptome, i.e., the complete set of RNA transcripts that are produced by the genome. These include tRNA, mRNA, rRNA, and a class of non-coding RNAs (e.g., miRNAs, IncRNAs, snRNAs, and snoRNAs). Subsequently, the transcriptome can be chemically modified, thus adding another layer of regulation. This far 1- and 6-methyladenine (m1A and m6A respectively) are known to modulate the transcriptome [54–56].

MicroRNAs (miRNAs) are small noncoding RNAs that mediate silencing and post-transcriptional regulation of gene expression [57]. miRNAs regulate processes such as cell differentiation, proliferation, migration, and apoptosis. They regulate gene expression by base paring partially complementary binding sites in the 3′-UTRs of their mRNA targets, resulting in translational silencing or mRNA degradation [57, 58]. Therefore, they prevent mRNAs from performing their function. Since miRNAs are potential targets for future HSCR treatment, identifying the miRNAs and their target genes is crucial. Sergi et al. summarized the miRNA studies to date exploring involvement of miRNA in HSCR [59]. Several miRNA target genes have been linked with HSCR, most of which are involved in cell migration and proliferation. Downregulation of miRNA-34b, miRNA146a, miR-196a2, miR-200a, miR141, and mi-R-192 and upregulation of miR-195, miR206, and miR-218-1 have been described [59]. These changes in miRNA alter expression of genes involved in the pathogenesis of HSCR. Li et al. [58] identified 50 experimentally validated miRNA targets associated with HSCR, and the results support a deregulation of RET in HSCR patients.

Two decades ago, scientists at Ohio University identified a remarkable increase, 8–15-fold, of the expression of the m6A methyltransferase following cellular transformation [60]. The reversion to a non-transformed state resulted in a reduction of the m6A methyltransferase activity. Today, we know that the m6A modification is reversible and open to dynamic regulation [61]. The enzymatic apparatus for methylating adenines (A) to m6A in mRNA (writers), for reading these m6A marks (readers) and reversing those (erasers), has recently been identified and characterized. The methyltransferase complex consists of two active methyltransferase components, METTL3 and METTL14, and recently, it was demonstrated that METTL3 is required for cortical neurogenesis. Methylation of A to m6A does not alter the stability or coding properties of adenine in mRNA. Thus, the role of m6A is accomplished by proteins specifically binding to mRNA containing m6A. Three major binding proteins are YTHDF1, 2, and 3, of which YTHDF2 is shown to modulate neural development in mice [62]. The reversible potential of m6A underscored the role of m6A and its modifiers in post-transcriptional regulation. Some of the AlkB homologs are known to function as erasers [63–66]. This class of enzymes is involved in general epigenetic regulation, including reversal of methyl modifications from mRNA and tRNA [67, 68]. Genome-wide association studies have linked variants of the FTO and ALKBH5 demethylases with neurogenesis, thus implicating m6A dynamics in neural development. In addition, ALKBH1, ALKBH3, and ALKBH8 are known to reverse RNA methylations [64, 69–72], yet their involvement in neural development has not been shown. However, the phenotype of Alkbh1^−/−^ animals indicates a role of ALKBH1 in neural crest specification [63]. Despite an increasing body of evidence in support of a crucial role of reversible RNA modifications in neurogenesis and development of the central nervous system, no studies have investigated the role of these modifications in ENS development.

## Future therapies

### Cell-based therapy

The prospect of stem cell therapy for regenerative medicine is a promising avenue for treating enteric neurocristopathies, such as HSCR in the future. Researchers now attempt to repopulate the aganglionic bowel of children with HSCR [73]. The ideal neuronal replacement therapy would be that transplanted cells come from the affected child itself, to avoid immune rejection. Following culture in vitro for expansion and differentiation of the cells, the patient’s cells can be reimplanted into the gut wall, migrate to the site of the endogenous ENS, and differentiate to neurons and glial cells [74], ultimately regenerating the missing ENS. Yet, many questions remain to be answered, such as what the best approaches are to select, harvest, isolate, expand, optimize, and transplant stem cells into the gut to ultimately restore gut function.

Several sources of stem cells have been considered as potential candidates for cell-based therapy, such as embryonic stem cells (ESCs), enteric neural stem/progenitor cells (ENSPCs), or induced pluripotent stem cells (iPSCs). The differentiated derivatives of stem cells (ENSPCs) are less pluripotent than the undifferentiated stem cells (ESCs). This is an advantage because they are more mature and thereby restricted to the ENS cell lineage. Hence, the possibility that they differentiate into unwanted phenotypes and form tumors is limited [75–77]. In addition, some of the technical and ethical concerns associated with ESCs are avoided. The most innovative source of cells is iPSCs, the cells that arise when differentiated cells are reprogrammed back into a pluripotent state. The advantage of using iPSCs is that they can be derived from the skin or blood cells of the affected children; thus, they would provide the most minimally invasive method of obtaining cells for transplantation [78].

Reproducible methods to harvest therapeutic cells and evaluate the functional outcome have been tested in established animal models of the disease and with human cells in vitro. Thus far, stem cells have been transplanted into animal models such as chick neural crest, embryonic chick hindgut, and postnatal aganglionic mouse colon. Stem cells have shown the ability to migrate, proliferate, and differentiate into neuronal subtypes and restore a normal pattern of contractility to the aganglionic bowel [79–83]. Interestingly, transplantation of ENSPCs prevented premature death of HSCR mice [75]. Moreover, p75-sorted cells from ganglionic segments of the resected colon of HSCR patients were co-cultured with aneuronal colon. These cells successfully colonized the originally aneuronal segment, where they proliferated and differentiated as neurons and glia [82]. To confirm that transplanted ENSPCs are able to induce muscle contraction when activated, the response to electrical stimulation has been measured in mouse models transplanted with human p75+ cells. Intracellular calcium increased as a response to stimuli, demonstrating that the transplanted cells can form electrically functional networks [83].

The harvesting of cells can be performed by minimally invasive techniques, and neural stem cells have been isolated by endoscopy from normal and aganglionic bowel of humans, and from neonates, children, and adults [79, 84]. The delivery of cells can be performed by ultrasound or endoscopically guided microinjections, either as a single injection into the intestinal wall or the peritoneum or as several injections along the intestinal wall [85–88]. The optimal site for injection has yet to be decided, as it will depend on the yield of cells and their ability to migrate. Future systematic studies using animal models are necessary to compare delivery methods, functional response, and integration with the host environment. Autologous stem cell therapy could replace surgical intervention as primary treatment for children with HSCR in the future [74].

Cell-based therapy involves a risk of tumorigenesis. Hence, long-term safety is of primary importance as this potential treatment is proposed for children with a long lifespan. It is unknown what happens when ENSPCs are transplanted back into the environment of a neonatal colon. Will they successfully integrate and become regulated by local mechanisms? Or will they continue to proliferate uncontrollably and form tumors? [77]. Moreover, does the ex vivo culture before transplantation change the cells genetically or epigenetically? [76, 89]. Mouse models of HSCR have been transplanted with ENCCs and followed up for 24 months to assess long-term safety. The transplanted cells migrated along the myenteric plexus, functionally integrated and did not give rise to tumors or spread to other organs [90]. Several strategies to avoid or minimize the risk of tumorigenesis have been suggested. One option is immunofluorescence labeling of the cells to observe their migration. Another is implementing the option of killing the cells after transplantation, namely by introducing an inducible apoptosis gene into the transplanted ENCCs so that all transplanted cells could be eliminated if neoplasia occurs [77]. In conclusion, several issues remain to be addressed when it comes to long-term safety [74, 76, 77].

For a new therapy to be feasible for patients, the methods for isolating ENSPCs must be improved, cells must be expanded to yield a sufficient amount of cells, and the cells must be delivered into the human bowel in a safe way [91].

### Drug-based therapy

Pharmacological approaches could be a potential therapeutic avenue for HSCR patients. One method is drug optimization of culture conditions of pre-transplanted cells in vitro; another method is drug optimization of the actual environment in which the cells are to be transplanted, namely HSCR bowel [74]. Hence, HSCR might be preventable in some genetically susceptible children.

The microenvironment of HSCR patients is hypothesized to be inhospitable for ENSPC colonization, thereby contributing to HSCR pathogenesis. Hence, transplantation of missing neuronal cells might not be sufficient to colonize the aganglionic gut. The addition of missing microenvironmental factors might also be required. Several studies have demonstrated the effect of such microenvironmental manipulation. Neurogenesis was induced when cells derived from human ganglionic bowel biopsies were cultured in the presence of GDNF [92]. Moreover, GDNF treatment of ENCCs resulted in increased expression of genes that are associated with terminal neuronal differentiation and synaptic signaling, and conversely reduced expression levels of genes involved in early neuronal differentiation and neuronal migration [93]. Similarly, the addition of retinoic acid to pre-transplanted ENSPCs might also be beneficial, as vitamin A deficiency has been found to contribute to ENS defects [94]. Also, co-transplantation of ENSPCs with 5-HT receptor agonists has been found to enhance neuronal density and proliferation [76, 95]. Moreover, ENSPC migration in vitro was enhanced when the protease BACE2 was inhibited. The gene that encodes BACE2 is located in the region of chromosome 21, and a duplication of this region increases the risk of HSCR. Also, trisomy 21 knowingly causes Down syndrome, a group of patients where the rate of HSCR is increased by 100-fold [74, 75].

In HSCR patients with aberrant epigenetic patterns, these could be potentially corrected pharmacologically [96]. Such epigenetic therapies are currently being used in cancer treatment, for example by inhibiting histone deacetylases (HDACs) and DNA methyltransferases (DNMTs). Two DNMT inhibitors have already been approved for other diseases, such as myeloid leukemia [97]. Moreover, it has been found that patients with breast cancer carcinoma have a pathologically hypermethylated EDN3 promoter area; hence, an efficient treatment could be to demethylate the EDN3 promoter [98]. Similarly, medical therapy could be used to manipulate dysregulated epigenetic patterns of HSCR-associated genes. For example, Griseri et al. found that a histone deacetylase inhibitor, sodium butyrate, rescued RET expression in lymphoblast cells derived from HSCR patients [99].

Lastly, reactivation of precursors that lie latent in HSCR intestine is also a hypothetical avenue for future treatment. Although ENS markers are not expressed in the aganglionic segment of HSCR patients, neuronal markers are expressed when cells from this aganglionic segment are cultured as neurosphere-like bodies (NLBs). This indicates that cells necessary to form a functional ENS are present, but inactivate. Hence, there is a possibility that these endogenous cells could be reactivated in situ to regenerate the missing ganglion cells in HSCR-affected bowel segments [88]. To activate these precursors in the distal colon, more studies regarding the developmental regulation of stem cells and more specialized precursor cells are required.

We must continue to explore both cell- and drug-based therapies for HSCR. A deeper understanding of ENS development, the interactions between the microenvironment and ENSPCs, and epigenetic regulation is required for researchers to be able to target potential goals for pharmaceutical treatment.

### Genetic manipulation

Because of the available modern technology CRISPR/CAS9, genetic manipulation must be mentioned as an option for future therapy. CRISPR/CAS9 is a powerful tool that potentially could correct monogenetic diseases, yet it could also benefit the more complex multigenetic disease such as HSCR. It enables us to correct gene-mutations associated with HSCR disease that influence neuronal cell proliferation, migration, and differentiation [91, 92]. CRISPR/CAS9 has been used to correct RET mutations in ENCCs, created from HSCR-patient biopsies, that had defects in migration and neuronal lineage differentiation. After gene manipulation, the ability of these ENCCs to migrate and differentiate in vitro was restored [92]. Hence, it is possible that CRISPR/CAS9 technology can enhance, if not completely restore, neural crest cell function. Moreover, it is possible to manipulate the regulators of genes, such as transcription factors which control neuronal differentiation and ensure neuronal diversity [100]. These have been demonstrated to be dysregulated in HSCR patients and could potentially be corrected by gene editing [101].

In addition to genetic manipulation of the genome, targeting of epigenetic and epitranscriptomic mechanisms may open avenues for novel treatment. Epigenome editing mediated by CRISPR/CAS9 means manipulation of gene transcription and expression without directly modifying DNA sequences [100]. Epigenetic treatment could correct disorders with aberrant epigenetic marks as the underlying pathophysiologic mechanisms. For example, one could mutate histones so they cannot be acetylated, either by manipulation of the acetyltransferases that transfer acetyl groups to the histones or by manipulating the target locus on histones, making it unable to bind the acetyl group. Similarly, one could induce methylation or demethylation on the DNA strand [100]. The promise of stem cell replacement therapy for HSCR could also benefit from epigenetic editing. Targeted epigenetic silencing or reactivation at a desired time point or cell stage has the potential to confine the direction of cell differentiation and yield sufficient numbers of the desired cell type for transplantation [102, 103].

With CRISPR/CAS9 technology lies a potential to advance both in basic and translational research. Manipulation of the genome and epigenome enables us to study the functional and biological role of genetic and epigenetic regulation. Thereafter comes the exploration of genome/epigenome editing-based therapeutics [103]. More studies are required to unravel how genetics and epigenetics influence ENS development and HSCR pathogenesis [92]. CRISPR/CAS9 is a promising tool to obtain this knowledge, as it has made it easier to set up in vitro studies of both animal and human cells, generate transgenic mouse models, and facilitate stem cell-based therapy for HSCR [103–105]. However, ethical and legislative aspects must be taken into consideration when using a genomic approach to explore new therapeutic options.

## Summary and conclusions

Knowledge of regulation in the development of the ENS and regenerative medicine to treat HSCR shows great promise at the pre-clinical level. A lot of research has been done in the field of stem/progenitor cells and cell-based therapy, yet these findings remain to be translated from the bench to the bedside. Significant questions remain to understand the complex etiology of HSCR, both regarding the genetic, epigenetic, epitranscriptomic, cellular, and molecular events of ENS development: How do epigenetic inheritance and environmental influences affect HSCR pathogenesis? How does the regulation of the chromatin landscape contribute? Can we ever fully understand the cross-talk that occurs between modifications? [106]. Together with ethical issues, these questions need to be resolved for cell-based or medical therapy for HSCR to progress toward clinical trials [9, 107]. Optimism and innovation in pediatric surgery in combination with solid progress in the laboratory will hopefully lead to a new treatment for children with Hirschsprung disease [106].

## Data Availability

Not applicable

## Abbreviations

DNMTs: Methyltransferases
ENCDCs: Enteric neural crest-derived cells
ENS: Enteric nervous system
ENSPCs: Enteric neural stem/progenitor cells
ESCs: Embryonic stem cells
ESCs: Enteric stem cells
HATs: Histone acetyltransferases
HDACs: Histone deacetylases
HMTs: Histone methyltransferases
HSCR: Hirschsprung disease
iPSCs: Induced pluripotent stem cells
m6A: 6-Methyladenine methyltransferase
MeCP2: Methyl binding proteins
miRNAs: MicroRNAs
NLBs: Neurosphere like bodies
